# Enhancement in Cathodic Redox Reactions of Single-Chambered Microbial Fuel Cells with Castor Oil-Emitted Powder as Cathode Material

**DOI:** 10.3390/ma14164454

**Published:** 2021-08-09

**Authors:** Shobha Suresh Kumbar, Dipak Ashok Jadhav, Chetan S. Jarali, Dhananjay B. Talange, Asif Afzal, Sher Afghan Khan, Mohammad Asif, Mohd. Zulkifly Abdullah

**Affiliations:** 1 Research Scholar, Visvesvaraya Technological University, Belagavi 590018, India; 2 Department of Electrical Engineering, Sanjay Ghodawat University, Atigre 416118, India; 3 Department of Agricultural Engineering, Maharashtra Institute of Technology, Aurangabad 431010, India; deepak.jadhav1795@gmail.com; 4 Structural Technologies Division, CSIR NAL, Bengaluru 560017, India; dr.chetan.jarali@gmail.com; 5 Department of Electrical Engineering, College of Engineering, Pune 411005, India; talanged@gmail.com; 6 Department of Mechanical Engineering, P.A. College of Engineering (Affiliated to Visvesvaraya Technological University, Belagavi), Mangaluru 574153, India; 7 Department of Mechanical Engineering, Faculty of Engineering, International Islamic University, Kuala Lumpur 53100, Malaysia; sakhan06@gmail.com; 8 Department of Chemical Engineering, College of Engineering, King Saud University, P.O. Box 800, Riyadh 11421, Saudi Arabia; masif@ksu.edu.sa; 9 School of Mechanical Engineering, Engineering Campus, Universiti Sains Malaysia, Nibong Tebal 14300, Malaysia

**Keywords:** catalyst characterization, cathodic reduction, electrochemical wastewater treatment, microbial fuel cells, power density

## Abstract

Microbial fuel cell (MFC) would be a standalone solution for clean, sustainable energy and rural electrification. It can be used in addition to wastewater treatment for bioelectricity generation. Materials chosen for the membrane and electrodes are of low cost with suitable conducting ions and electrical properties. The prime objective of the present work is to enhance redox reactions by using novel and low-cost cathode catalysts synthesized from waste castor oil. Synthesized graphene has been used as an anode, castor oil-emitted carbon powder serves as a cathode, and clay material acts as a membrane. Three single-chambered MFC modules developed were used in the current study, and continuous readings were recorded. The maximum voltage achieved was 0.36 V for a 100 mL mixture of domestic wastewater and cow dung for an anodic chamber of 200 mL. The maximum power density obtained was 7280 mW/m^2^. In addition, a performance test was evaluated for another MFC with inoculums slurry, and a maximum voltage of 0.78 V and power density of 34.4093 mW/m^2^ with an anodic chamber of 50 mL was reported. The present study’s findings show that such cathode catalysts can be a suitable option for practical applications of microbial fuel cells.

## 1. Introduction

Consumption of energy has been increasing day by day. Considering the resources available for the generation and their rate of depletion, alternative resources should be used. Usage of new energy sources efficiently, renewable energy, research in energy-saving techniques are important factors at present and for the future [1]. One of the renewable sources is MFC, which leads to the generation of green energy in the form of bioelectricity [2]. Electronic bacteria use the wastewater as a feed to convert biodegradable organic matter into electricity. Further, MFC can be integrated with wastewater treatment plants to make the process efficient and cost-effective [3]. Typical MFC consists of anode, cathode, and separator. There are different configurations of MFC viz, two-compartment, single-compartment, flow, and stacked [4]. The selection of electrode materials used for the construction matters greatly from the cost perspective and should have suitable conducting properties. Carbon forms such as graphene have suitable electrical conducting properties [5]. Nanomaterials, mxenes nanomaterials [6], multiwalled carbon nanotubes (MW-CNTS) [7], conducting polymers [8,9] and composites have the suitable potential for electrode materials as they offer optical, electrical, and biochemical characteristics [10]. In developments of electrode materials and their characteristics are important constraints for the long-term operation of MFC [11]. Exoelectrogens in anodic chamber release electrons to the anode and protons into the solution through anaerobic oxidation of biodegradable organics present in wastewater. The produced electrons are transferred to the anode and then to the cathode through an external circuit. Protons are transferred from anode to cathode through a cation exchange membrane internally, which separates both compartments [12]. The electron transfer mechanism is vital for charge transfer and energy recovery [13,14]. On the cathode, the electrons and protons combine with oxygen to form water. In experimental work, the external load was connected to anode and cathode and reported that the flow of electrons drastically reduced cell voltage from a maximum voltage of 1.2 to 0.3–0.8 V [15]. Performance analysis of 45 L pilot-scale microbial fuel cell (MFC) made from glass-fiber-reinforced plastic and ceramic-separators (CS) with multiple electrode assembly was carried out. A study on the effect of external resistance (Rext) varying from 100 to 3 Ω revealed that maximum power (Pmax) of 14.28 mW was obtained under the given operating conditions [16]. In another work, the highest power density achieved was up to 2.15 kW/m^3^ with an anodic chamber volume of 0.3 mL with the use of pure culture inoculums [17]. For the development of MFCs, instead of Nafion membrane, which is costly, a clayware ceramic separator was used as membrane and revealed a maximum power density (Pmax) of 16.8 W/m^3^ [18]. Coconut shell can also be used as a membrane in MFC applications and yielded two times more power density than Nafion 117 membrane [19]. Cross-linked polyvinyl alcohol has been proved as an adhesive for separative material [20]. The application of activated carbon powder and other nanomaterials to treat pharma-waste has been reported earlier [21,22].

Conventional technology such as anaerobic digestor of microbial electrolysis held membrane reactor is energy-intensive and requires skilled labor for operation and maintenance; also, such technology does not have the energy-producing capacity as compared to microbial fuel cells. Hence, we have selected single-chambered microbial fuel cells to overcome these limitations [21,22].

### Motivation and Objectives

In present cases, much effort has been put forward to enhance the rate of cathodic redox reactions. A cathodic performance is one of the limiting factors for enhancing the overall performance of MFC. The present study is a novel approach to synthesize the cathodic catalyst from waste castor oil effluent. Work on MFC using castor oil-emitted powder as a cathode material was not reported earlier for MFC applications. Therefore, this experimental work attempts to evaluate the performance of different MFCs developed with synthesized graphene from waste graphite as an anode material with steel mesh as substrate and castor oil-emitted powder as a cathode material. The waste castor oil collected from car servicing stations has been used to synthesize the catalyst powder. The approximate cost of such waste castor oil is around 20–30 Rs per liter, which is a cost-effective solution compared to different oils used for catalyst synthesis. The process cost of the graphene and castor oil powder is very less. Similarly, a single-chambered MFC has been developed at minimum cost.

## 2. Materials and Methodology

In the present work, miniaturized three single-chambered MFCs are developed using synthesized graphene as an anode and castor oil-emitted powder as a cathode material. The performance of low-cost MFC was evaluated by measuring voltage, power density, and current density. As the thin film has to be placed in the anodic chamber for a long time, polyvinyl alcohol (PVA) crystals are used as an adhesive for the thin film preparation. The adequate volume of the modules was kept constant (100 mL) to compare the performance. The performance of the synthesized graphene from waste graphite and castor oil-emitted powder for bioelectricity generation is studied. Graphs were drawn by taking the average daily readings concerning the duration keeping resistance fixed and the variable resistance the polarization curve was drawn.

### 2.1. Material of Characterization of Cathode Catalyst

Carbon nanoparticles were obtained from the emission of ignited castor oil and used as cathode material. Carbon nanoparticles were collected by scraping the layer from the steel plate placed on the flame of castor oil after half an hour of collection, i.e., after cooling to room temperature. The sample from the collected carbon nanoparticles was used for its characterization. Out of four figures of sample characterization, one each is taken. Characterization consists of Fourier transform infrared spectroscopy (FTIR) and scanning electron microscopy (SEM) analysis, and its resistance was measured.

**Figure 1:**
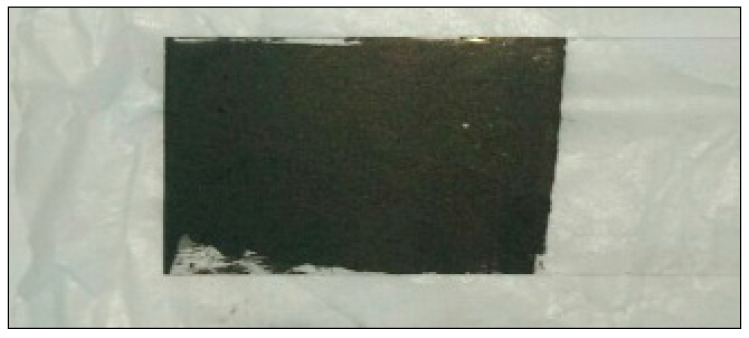
Thin-film of castor oil-emitted powder for characterization.

Resistance of the same powder was measured by preparing the thin film of the materials on a glass slide (substrate) using Rigol DM3058 digital multimeter. It was having a resistance of 196.76 Ω. The thin film developed is shown in Figure 1.

### 2.2. Electrode Preparation

**Figure 2:**
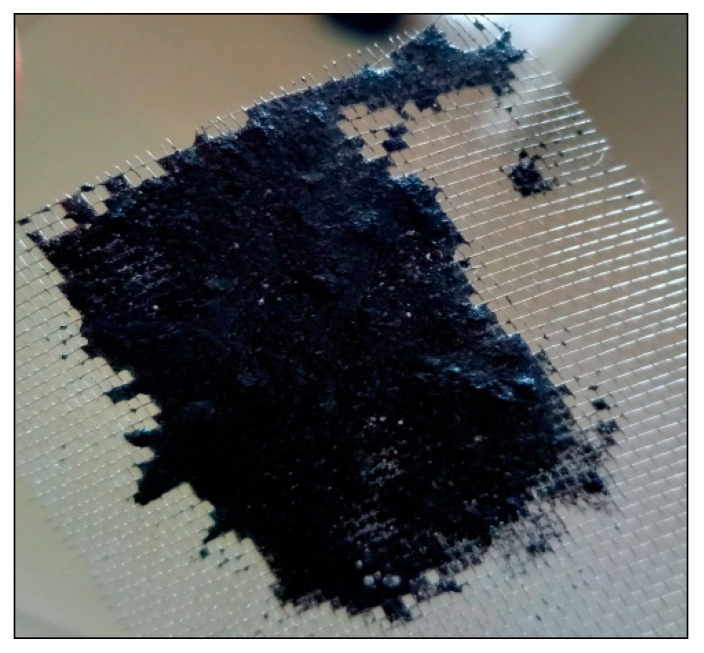
Stainless-steel wire mesh anode.

**Figure 3:**
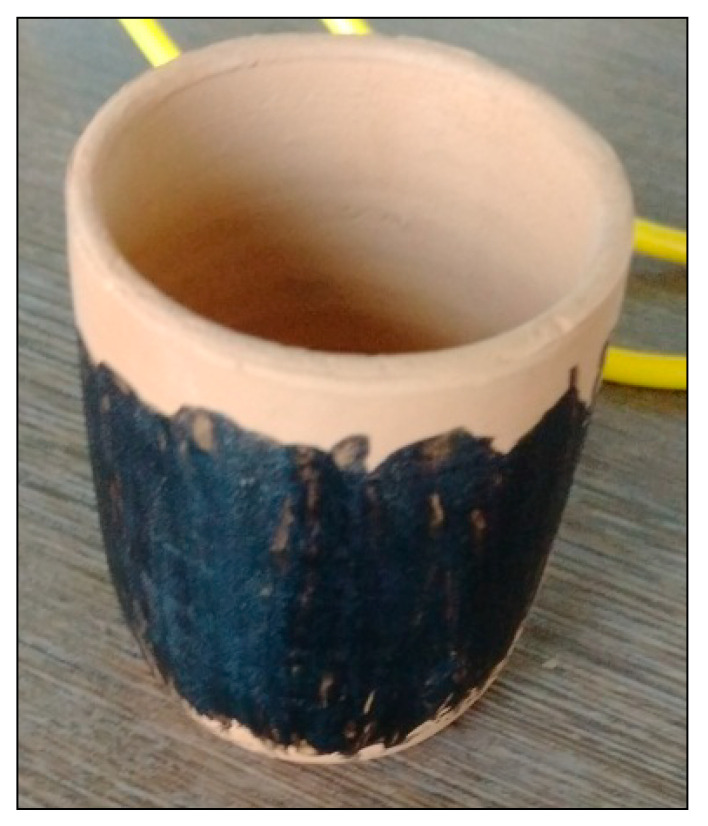
Ceramic separator with an electrode coated with graphene air-cathode assembly.

The anodic thin film was prepared by steel mesh and synthesized graphene. Synthesis of graphene from waste graphite and its characterization was reported earlier [23]. An anode electrode was prepared by the doctor’s blade method. Solution of 1 g of PVA with 10 mL of distilled water was prepared using a magnetic stirrer, then 5 to 8 drops of the solution prepared added to 1 g of graphene for steel mesh of 4 × 4.5 cm. Steel mesh obtained was kept in the oven for one hour at 180 °C temperature and cured, shown in Figure 2. The exact process was followed for the cathode preparation too. About 5 to 8 drops of the solution prepared was added to 1 g of castor oil-emitted powder and pasted on the clay pot. It was kept in the oven for one hour at 180 °C temperature for curing. It is shown in Figure 3.

### 2.3. MFC Set Up and Construction

**Figure 4:**
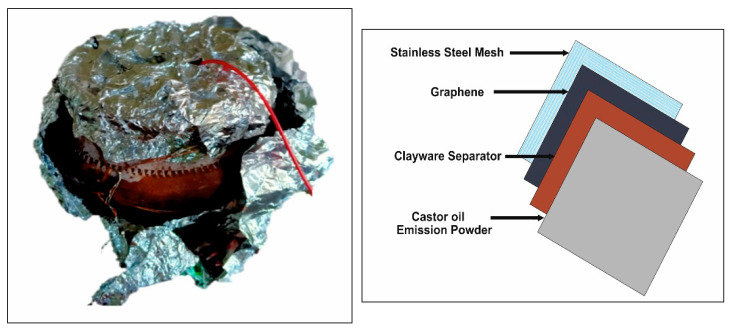
Configuration of MFC modules and separator electrode assembly.

Figure 4 illustrates the working model of the single-chamber MFC. For MFC1, clayware taken was 100 mL, and domestic waste used was 100 mL. MFC2 and MFC3 were of 100 mL. Details of different MFCs developed are mentioned in Table 1. As shown in Table 1, MFC4 is used at control MFC for comparison slurry as wastewater instead of inoculum.

A wastewater sample from the campus of Sanjay ghodawat group of institutions was used in the current study with chemical oxygen demand (COD) of 348–571 mg/L and biochemical oxygen demand (BOD) of 74–144 mg/L [24]. External resistance was connected between anode and cathode to measure the voltage using a multimeter. For calculation of parameters such as current, power, power density, volumetric power density, current density, volumetric current density following equations were used (Equations (1)–(6)).

For current,

(1) I=E/R

where *I* is current in mA, *E* is voltage in volts, *R* is resistance in Ω, *P* is power in mW, PD (power density) is mW/m^2^, VPD (volumetric power density) is mW/m^3^, CD (current density) is mA/m^2^, and VCD (volumetric current density) is mA/m^3^. Aa is the area of the anode in m^2,^ and Vac is the volume of the anodic chamber,

Power calculations,

(2) P=E×I

(3) PD=P/Aa

(4) VPD=P/Vac

(5) CD=I/Aa

(6) VCD=CD/Vac

Current (Equation (1)), power (Equation (2)), power density (Equation (3)), volumetric power density (Equation (4)), current density (Equation (5)) and volumetric current density (Equation (6)) are calculated by using average values of the readings noted daily.

## 3. Results and Discussion

In this section, the performance tests were conducted on four MFCs developed, and current, power, power density, volumetric power density, current density, and volumetric current density obtained using Equations (1)–(6) are discussed.

### 3.1. Characterization of Castor Oil-Emitted Powder

**Figure 5:**
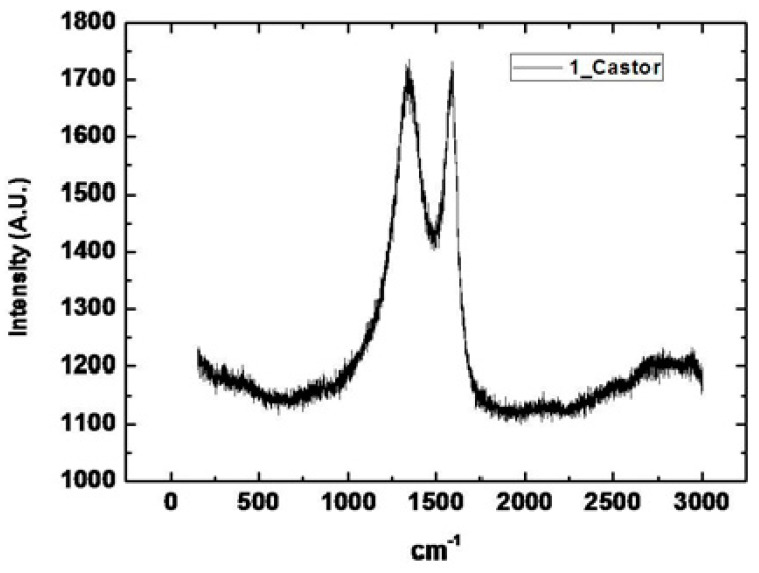
FTIR image of castor oil emission powder.

FTIR characterization was performed from Sophisticated Analytical Instrument Facility, Indian Institute of Technology, Bombay, using model 3000 Hyperion Microscope with vertex 80 (Bruker, Germany). FTIR image of castor oil-emitted powder has been given in Figure 5. FTIR graph having wave number observed at 1600 cm^−1^ exhibits the property of carbon. The peak observed in FTIR shows that strong carbon-carbon bonding helping for effective electron transfer in MFC.

**Figure 6:**
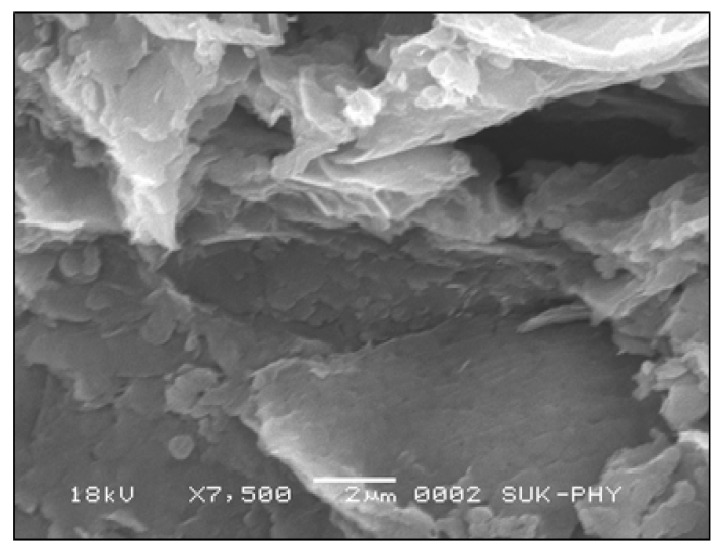
SEM image of castor oil-emitted powder.

Carried out with the Department of Physics, Shivaji University Kolhapur, SEM with EDS was performed by using Machine Make: JEOL Limited. Japan Model: JSM-6360 (JOEL Limited, Japan) and results are shown in Figure 6. It was observed that the castor oil-emitted catalyst showed porous structure, which can be useful for enhancing the overall surface area of the electrode cathode. The sharp peak at 1600 cm^−1^ is due to C–C bonding linearly adsorbed on a low coordinated metallic electrode surface located at the nanoparticles’ corners or edges. Moreover, another band at 1350 cm^−1^ is related to CO adsorbed on the bridge and hollow sites of the C–C ensembles exposing more active sides for cathodic reduction. Extensive characterization by multi-technique approach was performed to obtain detailed information on the role played by crystal phase, morphology, surface electronic states and coordination number of synthesized carbon particles and establish a proper size range to obtain the best catalytic performances in MFC.

### 3.2. Effect of External Resistance on Power Production

**Figure 7:**
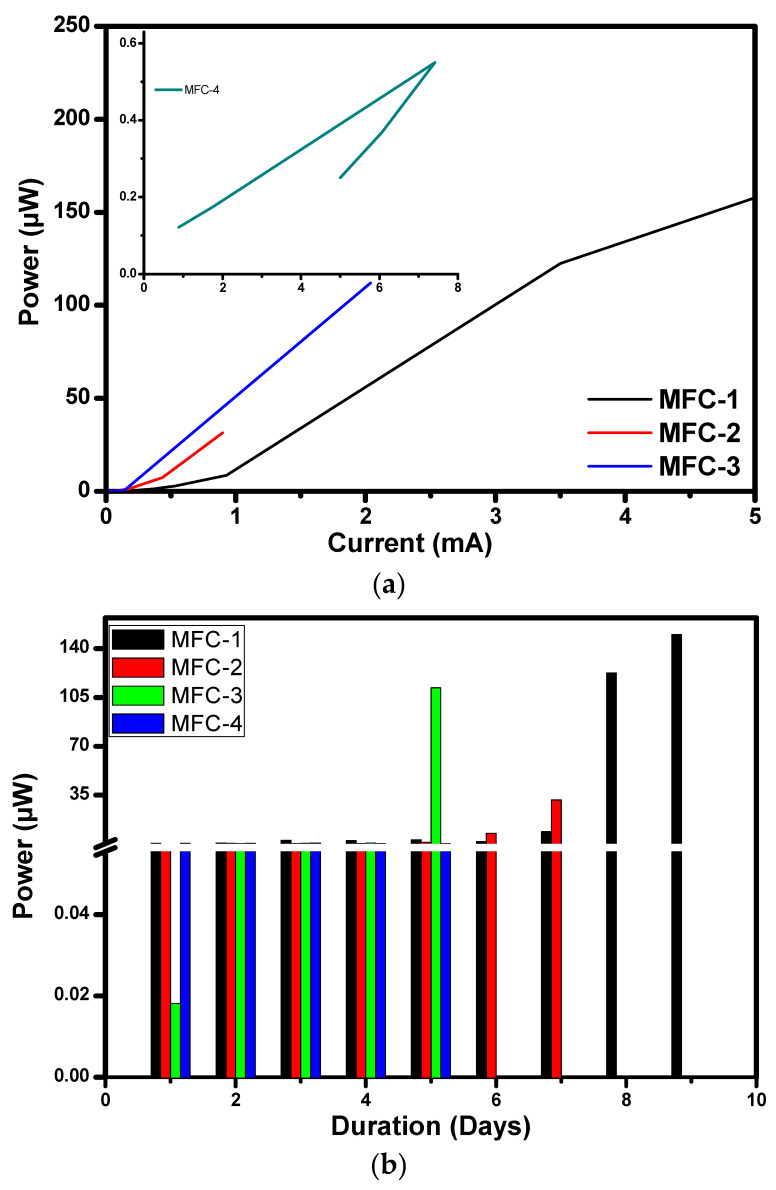
(**a**) Current vs. Power for all MFCs; (**b**) Graphical representation of duration vs. power.

The effect of duration on power, power density, and the current density is discussed in this section. Figure 7a explains the effect of current on different MFC modules and the effect of duration on power with fixed resistance as a load connected between anode and cathode in Figure 7b. Depicted values in the graphs are for MFC1 is of 9 days, MFC2 of 7 days, MFC3 and MFC4 of 5 days during the start-up phase of MFC operation.

#### 3.2.1. Effect of Current and Duration on Power on MFC Modules

Figure 7a,b provide variation in power concerning the current and duration of MFC1–4. Power generated from MFC1 increases from 0.000204 to 13.10 mW from day 1 to day 19 with external resistance of 10 Ω. During the first eight days, much less power generation was observed, and from day nine onwards, there was a very sharp increase in power generation. This could be the lesser release of electrons by the microbes during the first eight days, and more release of oxygen during the next successive days might be the reason for the result reported. The maximum value of power generated was 13.1 mW on the 19th day. Similar results were reported in the literature [17]. A separator made of red soil is suitable for MFC fabrication to generate higher power [25]. The power of MFC2 increased from 0.09 to 1.98 mW from day 1 to day 13 with external resistance of 39 Ω. During the first six days, much less power generation was observed, and from day eight onwards, there was a very sharp increase in power generation found. Compared to MFC1, power generation was noted on day 6, and on day 13, maximum power was generated. The reason for the increase in power could be an increase in voltage. Due to constant resistance, the current is dependent on the voltage. Voltage increase is based on the increase in electrons accumulation on the anodic film. Internal losses such as Ohmic losses, activation losses, concentration losses also matter a lot in power production.

Power generated for MFC3 increased from 0.02 µW to 3.4 mW with an external resistance of 27 Ω, from day 1 to day 12. During the first four days, much less power generation was observed, and from day five onwards, there was a very sharp increase in power generation found. The reason for the increase in power could be an increase in voltage. Voltage increased due to electrons accumulation on the anode and less resistance connected between anode and cathode. Compared to MFC2, voltage generation started early in the case of MFC3. The power of MFC4 increased from 0.03 to 0.06 mW and again decreased to 0.05 mW, as shown in Figure 7b. External resistance of 10 Ω connected to MFC4 from day1 to day5. The increase in power could be inoculums used, sugar cane slurry used to test the performance. Day 1 voltage generated in the MFC and continued till day 5.

Maximum voltage was generated by the cell compared to MFC1–3. In addition, the size and inoculums quantity used was less in MFC4. Further, volumetric power density and volumetric current density were calculated for four modules. The maximum volumetric power density obtained in MFC1 is 65.5 mW/m^3^, the volumetric current density is 181 mA/m^3^. MFC2 depicted volumetric power density 19.8 mW/m^3^, volumetric current density is 71.3 mA/m^3^, MFC3 achieved 34 mW/m^3^, and volumetric current density is 112.2 mA/m^3^. MFC4-depicted volumetric power density is 1.28 mW/m^3^, and volumetric current density is 1.6 mA/m^3^. Similar results were reported in the literature [26]. The higher performance in the present study is mainly due to the enhancement in the rate of cathodic reactions with the use of novel catalysts.

#### 3.2.2. Effect of Duration on Power Output and Current Output of MFC Modules

**Figure 8:**
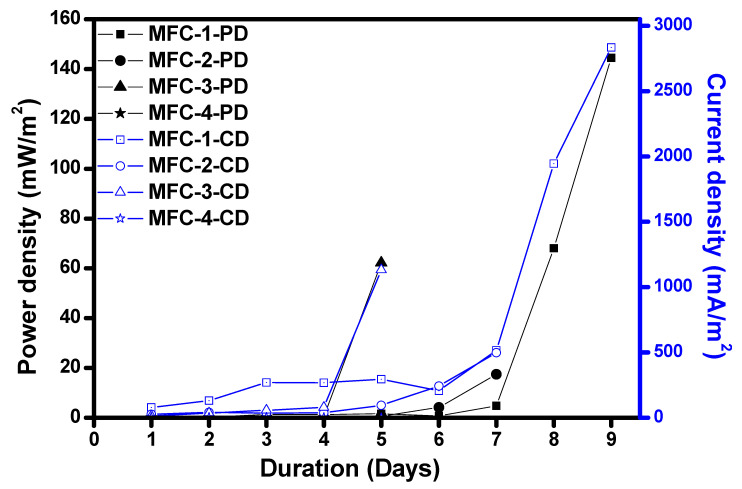
Duration vs. current density and power density for all the MFCs.

The effect of duration on power density, current density has been discussed in this section. Figure 8 explains the effect of duration on different MFC modules with fixed resistance as a load connected between anode and cathode.

Figure 8 illustrates the variation in power density and current density concerning the duration of MFC1–4. The power density of MFC1 increased from 0.11 to 158.22 mW/m^2^, and the current density was from 79.44 to 2811.11 mA/m^2^ between day 1 to day 19. The reason for this could be an increase in power generated. Similar results were revealed in the literature [27,28,29]. The current density increase could be the thick layer of bacteria on biofilm, which tries to overcome the surface of the anode by decreasing the rate of wastewater consumption by the biofilm [30]. This limits the power generation and sometimes the possibility of biofilms responsible for passing current to the surface.

In MFC2, power density increased from 0.05 to 1100.9 mW/m^2^, current density has increased from 27.35 to 3960 mA/m^2^, as shown in Figure 8 from day 1 to day 13. An increase in voltage could be the reason for this trend, and the voltage on day 1 was 1.9 mV and increased to 278 mV till day 13. In addition, the current from 0.049 to 7.12 mA till day 13. Due to an increase in voltage, power, power density, volumetric power density increased. Current density is increased with current, then volumetric current density.

Power density and current density of MFC3 varied from 0.01 to 1889.07 mW/m^2^, 14.4 to 6234 mA/m^2^ external resistance is 27 Ω as shown in Figure 8. Voltage generation increased from 0.7 to 303 mV, current 0.025 mA to 11.22 mA from day 1 to day 12. The volumetric power density of day 1 was 0.18 mW/m^3^, an increase to 3403.3 mW/m^3^ on day 12, and volumetric current density 259.2 mA/m^3^ of day 1 increased to 11,222 mA/m^3^ on day 12. The increase in voltage could be the increase in power, power density, and volumetric power density. Variation in current will vary the current density, volumetric current density.

In MFC4 power density increased from 13.89 µW/m^2^ to 0.63 mW/m^2^ and decreases to 28.24 µW/m^2^. The current density reported 27.78 µA/m^2^ to 0.49 mA/m^2^ with an external resistance of 10 Ω, as shown in Figure 8. The day on which the maximum power, power density, current density, volumetric power density, volumetric current density was achieved is reported in Table 2.

### 3.3. Effect of External Resistance on the Performance of MFC

**Figure 9:**
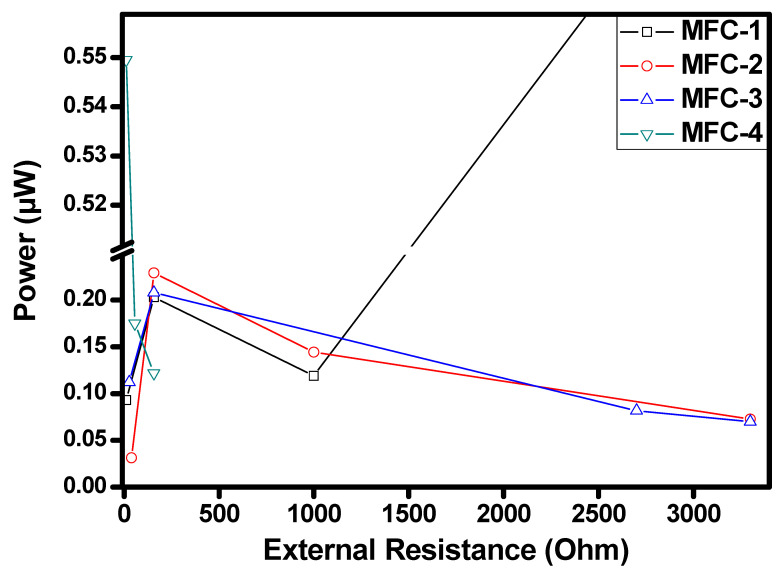
External resistance vs. power for MFC1.

The effect of external resistance connected between anode and cathode on power with respect to different MFC modules is illustrated in Figure 9.

At 10 Ω, power generated was 0.61 µW, and at 156 Ω, power generated was 1.2 µW. In addition, at 1000 Ω, 2.24 µW power was generated, and at 3300 Ω, power generated was 1.83 µW. It is found that as external resistance increases, power generation also increases to a certain extent. Drop in the power generation may be due to the losses existing in the cell, viz., Ohmic losses, activation losses, concentration losses [30,31,32]. Ohmic losses are resistance to the flow of electrons or the flow of ions through membranes and electrolytes. Activation losses occur during the transfer of electrons from or compound reacting at the electrode surface. Concentration losses are the limitation caused by the rate of mass transport of a species to and from the electrode surface. Power generated by the MFC2 after adding different resistances is also shown in Figure 9. At 39 Ω, 0.56 µW power was generated. A total of 156 Ω generated power 0.35 µW, at 100 Ω and 3300 Ω power generated was 0.44 µW and 0.37 µW. As external resistance increased, variation in the power generation was found. The reason could be that 39 Ω voltage generated was 0.2 to 0.4 V, but current decreased from 2.8 to 0.9 mA. However, as current increases, a decrease in cell voltage is observed due to increased internal losses [33,34]. In MFC3 at 27 Ω, power generated was 0.71 µW, at 156 Ω, it was 0.33 µW. In addition, 0.91 µW generated by 2700 Ω and 3300 Ω generated power 0.26 µW. The voltage generated at 27 Ω was 0.31 V, increased to 0.52 V at 3300 Ω with a 2.3 to 0.5 mA current. As external resistance increases, power generation also increases to a certain extent. Power generated by the MFC4 after adding different resistances is shown in Figure 9. It was seen that at 10 Ω, 156 Ω, and 56 Ω power generated 0.06, 0.001, and 0.01 µW, respectively. The primary reason for the significant drop of power for MFC4 with the increase in external resistance is the insufficient availability of inoculum for anodic oxidation as MFC4 contains only slurry having lower food to Microorganism ratio. Such a decrease in power is due to non-turnover conditions for electrochemical redox reactions and a decrease in the current [35,36,37].

Further, in MFC1, the voltage increased concerning resistance, i.e., from 0.31 to 0.35 V, resistance was 10 Ω in an initial stage and increased up to 3.3 KΩ, and the increase in power was observed. The voltage of MFC2 is increased from 0.2 to 0.4 V with a change in resistance from 39 Ω to 3.3 KΩ. MFC3 voltage is 0.71 to 0.9 V, resistance initially was 27 Ω and increased to 3.3 KΩ. It is observed that output voltage increases with resistance. 

## 4. Conclusions

In the present work, clayware as a separator, synthesized graphene, and castor oil-emitted powder for the anode and cathode, respectively, were used to reduce the overall cost of MFC developed. Four MFC modules were developed, and readings were recorded continuously to evaluate the performance. From detailed analysis following conclusions were drawn:

1. MFC1 with a larger anodic chamber and content generated a higher voltage of 362 mV compared to other models. The maximum power density of 145 mW/m^2^ is calculated. The maximum volumetric power density of 1.3 W/m^3^ and maximum volumetric current density of 2.8 A/m^2^ were achieved by MFC1.
2. Though MFC2 and MFC3 had the same anodic chamber volume and content, MFC3 generated a voltage of 303 mV, 8.8% more than MFC2. Power density and volumetric power density are 71.59% more than MFC2. MFC3 achieved a current density and volumetric current density of 57.43% more than MFC2.

MFC4 with inoculum slurry recorded a maximum voltage of 0.78 V and power density of 0.68 mW/m^2^ with an anodic chamber of 50 mL. The maximum current density of 0.49 mA/m^2^ was calculated. The maximum volumetric power density of 2.42 mW/m^3^ and maximum volumetric current density of 17 mA/m^3^ were achieved by MFC4.

Overall, it could be concluded that the higher the anodic chamber volume, the higher will be the power generated, keeping the electrolyte volume the same. MFC with red color separator yielded maximum power as compared to the red and white color separator. In addition, MFC developed with low-cost materials such as castor oil-emitted powder, synthesized graphene from waste graphite, and red soil clayware pot could be a viable clean and suitable for rural electrification with minimum cost. The present study’s findings show that such a cathode catalyst can be a suitable option for practical applications of microbial fuel cells.

## Figures and Tables

**Table 1:** Details of different MFC modules developed.

MFCs	Membrane	Wastewater	Inoculum	Effective Volume of Anodic Chamber
MFC1	Red color clayware	Domestic (50 mL)	Cow dung (50 mL)	100 mL
MFC2	Red+white color clayware	Domestic (50 mL)	Cow dung (50 mL)	100 mL
MFC3	Red+white color clayware	Domestic (50 mL)	Cow dung (50 mL)	100 mL
MFC4	Red color clay ware	Slurry (50 mL)	_	50 mL

**Table 2:** Comparison table indicating maximum parameters by modules.

Module	MFC1	MFC2	MFC3	MFC4-Slurry
External resistance in Ω	10	39	27	10
No. of days readings noted	10	13	12	05
Volume of anodic chamber in mL	100	100	100	50
Max voltage in mV	362 ± 22	278 ± 17	303 ± 25	787 ± 32
Power density in mW/m^2^	145	17	62	0.68
Current density in mA/m^2^	2833	499	1131	0.491
Volumetric power density in mW/m^3^	1301	314	1120	2.42
Volumetric current density in mA/m^3^	25500	8974	20370	17.1

## Data Availability

The data presented in this study are available upon request from the corresponding author.
